# Causal networks guiding large language models: application to COVID-19

**DOI:** 10.1007/s10729-025-09724-8

**Published:** 2025-10-13

**Authors:** Farrokh Alemi, Kevin James Lybarger, Jee Vang, Yili Lin, Hadeel R. A. Elyazori, Vladimir Franzuela Cardenas

**Affiliations:** Department of Health Administration and Policy, George Mason University, Fairfax, VA USA

**Keywords:** Large language models, COVID-19, Regression, Retriever augmented generation, Missing values

## Abstract

In the context of diagnosis of COVID-19, this paper shows how to convert a Causal Network to a Large Language Model (LLM). The Causal Network was converted to the language model using prompts and completions. Prompts were composed from the full-factorial combination of the text associated with statistically significant variables in the Causal Network. Completions were based on the evaluation of the probability of COVID-19 using the Causal Network. The accuracy of the Causal Network and LLM was tested using two databases. The first database was based on a survey of 822 patients, collecting 12 direct (parents on the Markov blanket of COVID-19 diagnosis node), 7 indirect (associated with COVID-19 but not direct cause) symptoms of COVID-19. The second set was based on 80 patients reporting their symptoms in open-ended questions, often reporting some of the direct predictors and rarely reporting any indirect predictors of COVID-19. The accuracy of Causal Network and Markov blanket was tested using Area under the Receiver Operating Curve (AUROC). When indirect information was available, the Causal Network model (AUROC = 0.91) was significantly more accurate than the LLM (AUROC = 0.88), even though LLM model was trained to duplicate predictions of the Causal Network. Where the indirect information was not available, both models had lower accuracy (AUROC of 0.75 and 0.76). The accuracy of LLM depends not only on patterns among direct predictors of the outcome but also data not reported to the LLM. Conversational LLMs need to go beyond information the patient supplies and proactively ask about missing, typically indirect, information.

## Introduction

At onset of an infectious pandemic, often information about gold standard lab test, the exact diagnosis, treatment and transfer mechanism of the infection are missing. Early on, clinicians notice unusual presentation of symptoms, which can be used to diagnose the infection – even when the diagnosis has no official name. If there was a way to use these symptoms to diagnose the patient, then triage decisions can be made in a manner that reduces the rapid expansion of the pandemic [1, 2]. Unfortunately, diagnosis of infections from combination of symptoms is complex and clinicians and patients need decision aids to make informed decisions. This paper provides an example of how a conversational AI system can help early diagnosis of infections at home, where predominantly only symptoms are available.

We demonstrate the ideas through diagnosis of COVID-19 at home, enabling interpretation of home tests, facilitating triage of the patient, encouraging the patient to follow tailored isolation protocols, and eventually through these steps, reducing the rate of re-infection and thus controlling the epidemy. To date, much progress has been made in developing systems for diagnosis of COVID-19 [3–7], but all of existing approaches rely on machine learning models. One needs to convert these algebraic models to a language model, before it can be accessible as a conversational AI system to patients at home. The purpose of this paper is to show how these conversions are done and whether, and when, the conversion leads to a loss in accuracy of the machine learning model.

For diagnosis of COVID-19, we focus on a Causal Network developed by our research team [8–12]. In these papers, we have shown that the model uses different symptoms for patients at different ages. The order of occurrence of symptoms matters, with some symptoms occurring early in onset of COVID-19 and others occurring after the third day of illness. The network model showed that symptom clusters mattered and no single symptom was sufficient for the diagnosis of COVID-19. The resulting network model was too complex for use by unaided clinicians and too inflexible for direct use by patients at home. Progress in AI has allowed natural language interactions with patients that could make it easier for patients to use the Causal Network model. In this paper we report our experience with converting the existing Causal Network to a conversational AI system.

A variety of analytical models are used to facilitate diagnosis [13–15] or treatment [16–20] decisions. We focused on the Causal Networks because in contrast to other machine learning techniques, it has an explicit procedure for addressing missing value issues [21, 22]. Causal Networks are chains of multivariate regression models, in which the values of each node in the network are LASSO regressed on all preceding events/variables [23]. The central regression equation in the Causal Network is the equation that predicts the outcome, in our case, diagnosis of COVID-19. All other equations impute the predictors of COVID-19, when these predictors are missing. Thus, a Causal Network transparently includes all associated imputation models.

In a language model, missing values are ignored. In conversations with LLMs, patients typically report what they have, and do not mention variables or symptoms they do not have. In contrast to language models, in Causal Networks, missing values are imputed. In these models, variables are divided into direct and indirect predictors. Direct predictors are “Parents in the Markov Blanket” of the outcome of interest [24]. When all direct predictors are reported, no unreported variable matters. It follows from the very definition of Markov blanket that indirect predictors are irrelevant in these circumstances. When the patient does not mention a direct predictor, then the situation is different. Then, indirect predictors are relevant and can be used to impute the missing direct variable. A contrast between Causal Network and language models helps clarify the role of indirect variables in improving accuracy and generalizability of language models.

## Methods

### Methods for deriving the causal network from data

**Fig. 1:**
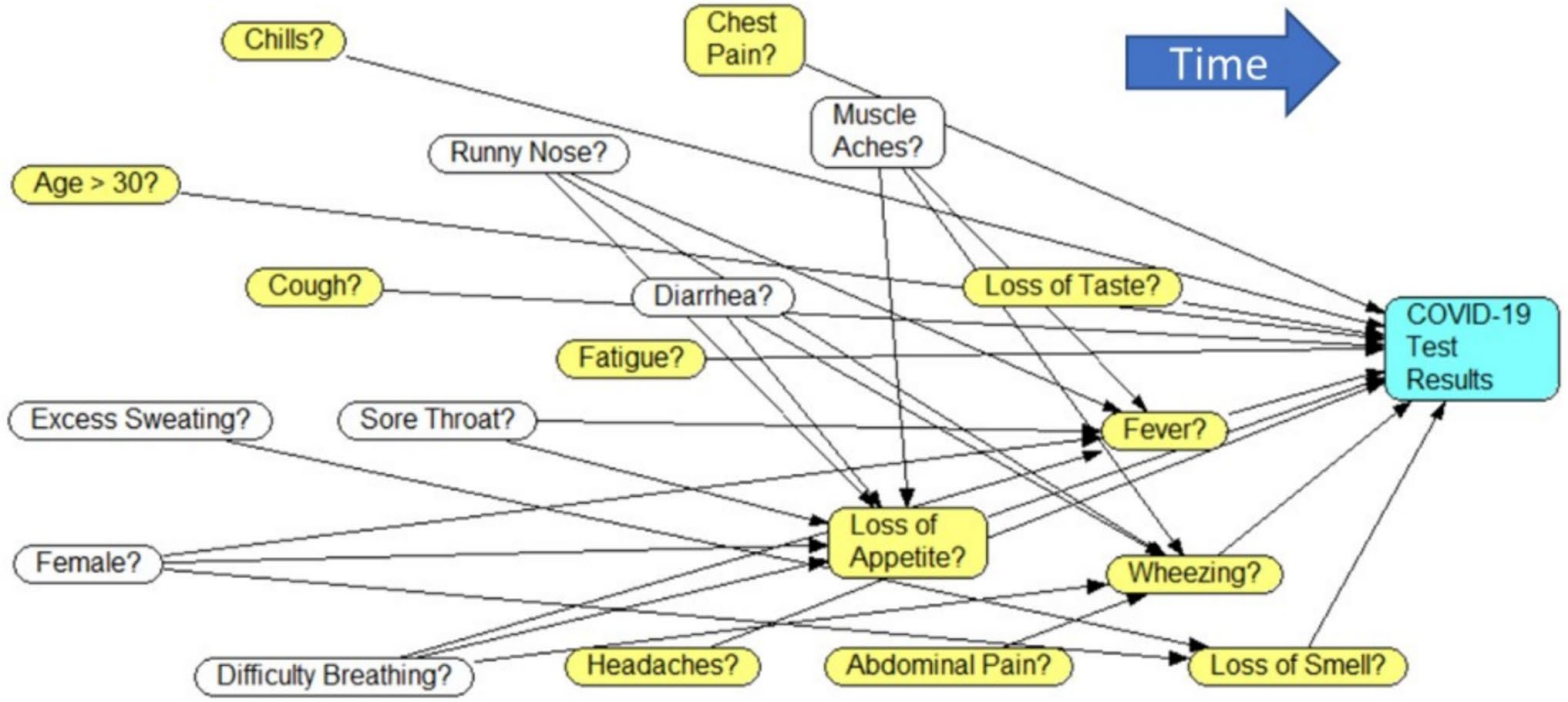
A Causal network for diagnosing COVID-19 from its symptoms, age, and gender. Figure Notes: Variables on the left are earlier events. Yellow nodes show a direct impact on the diagnosis of COVID-19. White nodes show indirect effects

As mentioned earlier, Alemi and colleagues surveyed 822 patients. They analyzed these data to construct a Causal Network to predict COVID-19 PCR test results from the patients’ symptoms. Colley’s method was used to assess the temporal order of occurrence of symptoms [25]. A chain of Least Absolute Shrinkage and Selection Operator (LASSO) regressions identified 19 robust, non-zero associations between symptoms and COVID-19 PCR test results. Subsequent regressions predicted the symptoms with non-zero coefficient in the LASSO regression, with symptoms from prior time points used to predict those at later time points. The resulting chain of regressions formed a causal network (see Fig. 1).

In Fig. 1, the progression of time is evident from left to right, with early symptoms like cough and later symptoms like fever. Direct contributors to COVID-19 diagnosis include: (1) age, (2) chills, (3) chest pain, (4) cough, (5) loss of taste, (6) fatigue, (7) fever, (8) loss of appetite, (9) wheezing, (10) headache, (11) abdominal pain, and (12) loss of smell. Indirect variables associated with COVID-19 were: (1) runny nose, (2) muscle aches, (3) diarrhea, (4) excess sweating, (5) sore throat, (6) gender, and (7) difficulty breathing. These indirect variables impute the values of the direct symptoms, when the direct variable is not reported by the patient.

### Methods of generating prompts and completions from causal networks

To train a language model to operate like a Causal Network, one can use prompt and completions. Separate prompts and completions are generated for each variable in the Causal Network. Prompts, and completions, must cover the entire range of each variable. Both language and network models rely on variables/nodes. Causal Networks can include continuous, categorical, and binary variables. Language models can process categorical or binary variables as text but have difficulty putting continuous variables into words. Continuous variables must be discretized, so that text can be attached to levels of the variables [26]. The simplest method of discretizing a monotone variable is to break it into three levels: Present, Absent, and Unknown. Non-monotone variables must be divided into monotone ranges, before discretizing. Once discretized, then each value of the variable is assigned a word or phrase. If a continuous variable is broken into categories, then the level of the discretized variable is assigned to the category labels.

After discretization, a prompt is constructed from all possible combinations of the variable levels. LLMs can be used to construct a description of the patient from a set of variables. Here is an example of prompts generated for diagnosis of COVID-19 from its symptoms and the corresponding completion (where the word probable is assigned to a probability of 0.64):

- **Prompt:** “Patient is 18 to 30 years old. It has been more than 3 days since onset of symptoms. Patient presents with chest pain, loss of appetite, loss of taste, loss of smell, and no other, relevant, late-stage, symptom. No home test reported.”
- **Completion:** COVID-19 is probable. Out of 100 persons in a similar situation 64 had COVID-19.

To generate all possible combinations of direct predictors of COVID-19, one uses a full factorial design, with some exceptions. In full factorial design, each level of the variable is combined with distinct levels of all other variables. If “n” variables have three possible levels (absent, present, and unknown), then a full factorial design produces 3^n^ combinations. To see how a factorial combination is constructed, consider if we had only three symptoms: loss of smell, excess sweating, and female gender. Table 1 shows all the possible combinations.

**Table 1:** Full factorial unique combinations of three variables

Prompt Number	Sex at Birth	Excessive Sweating	Loss of Smell
1	Female	Yes	Yes
2	Female	Yes	No
3	Female	Yes	Unknown
4	Female	No	Yes
5	Female	No	No
6	Female	No	Unknown
7	Female	Unknown	Yes
8	Female	Unknown	No
9	Female	Unknown	Unknown
10	Male	Yes	Yes
11	Male	Yes	No
12	Male	Yes	Unknown
13	Male	No	Yes
14	Male	No	No
15	Male	No	Unknown
16	Male	Unknown	Yes
17	Male	Unknown	No
18	Male	Unknown	Unknown
19	Unknown	Yes	Yes
20	Unknown	Yes	No
21	Unknown	Yes	Unknown
22	Unknown	No	Yes
23	Unknown	No	No
24	Unknown	No	Unknown
25	Unknown	Unknown	Yes
26	Unknown	Unknown	No
27	Unknown	Unknown	Unknown

In Table 1, the prompt generated for the first row could be: female patient has excessive sweating and loss of smell. Each row shows a different prompt. Row 4, for example, describes a female patient with loss of smell. No information is provided about excessive sweating. For another example, row 26 shows a patient who is able to smell and no other information is available. For still another example, the prompt in row 27 describes a patient in which none of the three pieces of information are available about the patient. There are a total of 27 row and 27 prompts in Table 1. Once the prompts are established, then completions are generated by evaluating the regressions at levels specified in the prompt; and assigning a word that corresponds to the estimated risk of COVID-19 [27].

In a full factorial design, the number of possible combinations grows exponentially as more variables are added. There are several ways to manage this exponential growth of prompts. One method is to rely on LASSO regression to minimize the number of direct predictors of COVID-19, thus reduce the combinations needed. Another method is to restrict the factorial combination to the observed cases within the data. Most data do not include all possible combinations of variables as some combinations are rare. In most data collection, 10% to 20% of the cases are repeated multiple many times and remaining cases are repeated few times. By directly relying on the data for prompts, the total number of prompts needed is reduced. A third method is to reduce the prompts by ignoring irrelevant variables in design of the full factorial combination. When all direct variables are specified, then indirect variables become irrelevant. In these circumstances, the indirect variables are irrelevant as direct variables are blocking their effects on the diagnosis of COVID-19. For instance, in Fig. 1 network, loss of smell blocks pathways from excessive sweating or gender to COVID-19 diagnosis. Consequently, some combinations of the three variables in Table 1 can be discarded when the value for loss of smell is known. This reduction transforms 27 full factorial prompts/rows in Table 1 to 11 prompts/rows (9 when loss of smell is unknown and 2 when it is known). In the COVID-19 Causal Network model, there were 12 direct and 7 indirect symptoms. A full factorial combination of variables provides for 3^19^ = 1,162,261,467 prompts and completions. After accounting for the exceptions, one needs only 3^12^ = 531,441 prompts and completions, if we assume one indirect predictor for each unknown direct predictor.

The method of generating prompts and completions leads to a structured specification of the prompts. Of course, patients do not follow the structure in these prompts. Language models match patients unstructured words to concepts in the structured prompts. For instance, while our structured prompt may expect the patient's sex, a patient may response:"I was born male but now am non-binary". The patient response requires the language model to classify the patient's sex at birth from the unstructured input. This type of understanding, often referred to as tagging or classification, is a strength of language models. Language models are quite accurate in these classification tasks, thanks to their extensive training on vast amounts of text.

In natural language, the order of words in a text is informative, as each prior word provides the context of subsequent words. The structured prompts derived from the regression equations require the input to arrive in the order specified in the prompt. This requirement can present challenges when applying them to language models, as the sequence of input may not always align with the model's expectations. If one assigns a word to each independent variable, then regression, and by extension the Causal Network model, and the prompts derived from it are essentially a bag of words, the order of variables does not matter. In contrast, in language the order matters. To say that the patient has “fever and chills” is not the same as saying that the patient has “chills and fever”. The former evaluates chills in the context of fever, perhaps saying that fever is high enough to lead to chills, a common situation, and the latter means something else, perhaps saying that fever came after chills. To train language models using structured prompts, we would need to construct prompts for all possible ordered combinations—an impractical number. To remedy this, the language model must be instructed to pre-process the input and organize it in the order expected in structured prompts. The reliance of language on order of words is one reason it is complicated to translate formulas into if–then rules needed for prompts and completions. Re-organizing the words into the expected order could address this difficulty. The expected order of the words was anticipated from the Casual Network model. Causal Networks are acyclical-directed graphs, where causes occur prior to effects. The arcs in these graphs provide an order among the variables. When users provide information outside the expected order, the system adjusted the phrases to revert to the expected order in the Causal Network.

### Unstructured testbed

We collected patient-reported COVID-19 symptoms through a set of web-based open-questions sent to 80 faculty at George Mason University. Consented respondents were asked to anonymously share their age, gender, symptoms, testing, and COVID-19 test outcomes using unstructured text.

In annotation phase, we identified features of interest from the Causal Network illustrated in Fig. 1, which included various symptoms and COVID-19 test results. Using the Doccano open-source annotation tool, the features of the Causal Network were annotated as a sequence tagging or entity recognition task. For the reliability of our annotations, two graduate students double labeled the free-text survey responses with the features that are present or experienced by the participant. This process ensured that our dataset accurately reflected the lived experiences of the participants. The inter-rater agreement was calculated at two levels: i) *span-level* – two annotations were considered equivalent if they shared at least one overlapping word and the same feature label, resulting in an F1 score of 86%, and ii) *document-level* –annotations were assessed based on a one-hot encoding of the presence or absence of each feature without considering the annotated span, yielding an overall agreement of 87%. Discrepancies between annotators were resolved by a third graduate student through an adjudication process.

The unstructured annotated text was used to evaluate the accuracy of the Causal Network and the LLM. We assessed the models'performance using the AUROC. This measure reports the trade-off between sensitivity and specificity of the two models in predicting PCR test results for COVID-19.

## Results

In tenfold cross-validation, both the regression models within the Causal Network and the language model constructed from its prompts achieved similar AUROC scores: 0.91 and 0.88, respectively (See Table 2). These findings indicate that performance of the language model deteriorates, even when both the language and the network models have the same word-order-restricted input.

**Table 2:** Area under receiver operating curve for language and causal network

Test Data Set	Language Model	Causal Network
822 patients surveyed for direct & indirect COVID-19 symptoms	0.88	0.91
80 unstructured descriptions of symptoms related to COVID-19	0.76	0.75

**Fig. 2:**
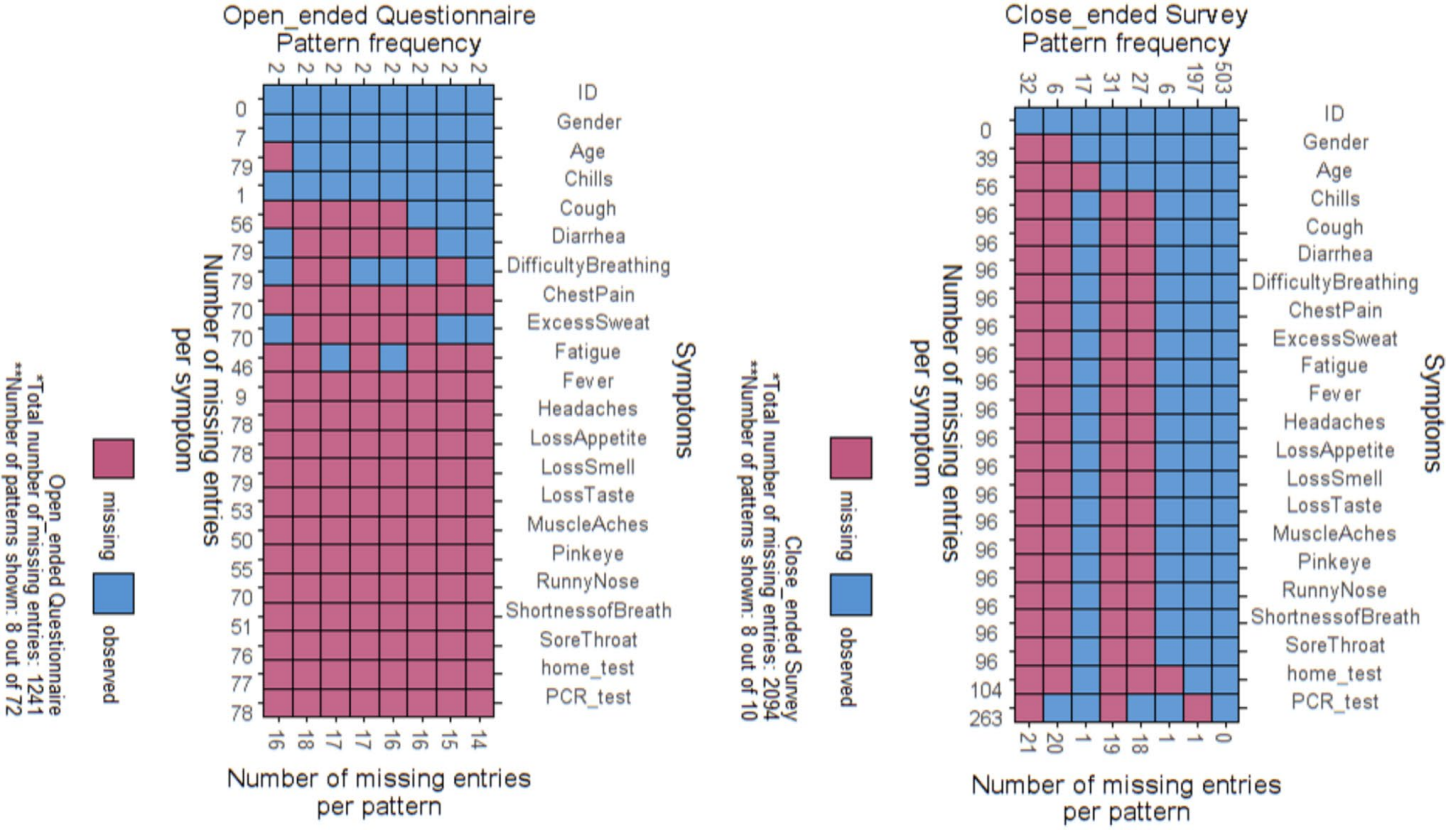
Patterns of missing symptoms in LLM and two testbed

Figures 2 illustrates differences in missing patterns of symptoms when using patient-specified prompts. Notably, open-ended questions showed more missing values compared to closed-ended structured surveys, even though the latter were longer. Some variables are never mentioned in the open-ended questions, possibly due to the small sample size or patients not considering certain variables crucial for COVID-19 diagnosis. The Causal Network has a built-in imputation to address missing values but in the unstructured text responses to open ended questions many of the variables needed for imputation of missing values were also missing.

In the close-ended questions with menu responses, there were 2,094 missing values in responses of 822 patients on 19 variables. In contrast, in the open-ended questions with unstructured text responses there were 1,241 missing values in responses of 80 patients on same 19 variables. Open-ended questions had 82% missing values while close-ended questions had 13% missing values. In the unstructured responses, indirect symptoms had extensive missing values. The rate with which indirect symptoms were not reported was 99% for diarrhea, 99% for difficulty breathing, 95% for sore throat, 88% for excessive sweating, 88% for runny nose, and 63% for muscle aches. Among indirect variables, only gender had a low rate, missing in 9% of cases. The language model had an AUROC of 0.76, while the Causal Network had an AUROC of 0.75.

## Discussion

This paper has shown a procedure for learning prompt and completion from Causal Networks. The application of this procedure to the diagnosis of COVID-19 produced a set of structured prompts. Training the LLM with these structured prompts led to a relatively high accuracy rate for both the LLM and the Causal Network. These data suggest that it is possible to convert a Causal Network into an LLM and the loss of accuracy is relatively small.

One reason Causal Networks can be converted to LLMs is because LASSO regressions used in constructing Causal Networks minimize the number of variables tracked, making it practical to construct prompts from full factorial combination of the variables. Markov Blankets reduced the number of prompts and completions needed. In addition, regression equations used in Causal Network construction enabled estimation of completions for the designed prompts.

When the Causal Network and the LLM were tested on unstructured text, the performance of both models deteriorated. Unstructured text response to open-ended questions had more missing values (82%) than structured close-ended questions (13%). In practice, lengthy surveys increase the risk of respondent prematurely abandoning the survey, leading to list-wise missing values. Open-ended questions, by allowing patients to mention only relevant variables, reduce survey length but introduce other sources of missing values as patients mention only what has occurred, and do not report what has not occurred. Of course, when needed information is not available, the performance of models deteriorates. Thus, one should expect poorer performance on open-ended questions for both models. Causal Networks, however, have built in mechanism for imputing direct predictors of outcomes from indirect predictors. At least theoretically, these models should have adjusted for missing values. In our data this was not the case. One possible explanation is that both the indirect and the direct symptoms had high rates of missingness in our data, preventing the Causal Network for correcting for the missing value problem.

To correct the problem of extensive missing values in unstructured text, we suggest that LLMs should directly ask for certain missing values during the conversation. An LLM conversation is more than an open-ended question. The machine has the capability to ask for a subset of missing values that could change the advice to the patient. In long conversations, typically a dialogue manager decides the next topic [28–33]. This paper suggests that dialogue managers can decide on the next topic by examining missing information needed for inference, as specified by the Causal Network. Causal networks specify the direct and indirect predictors of the outcome. If the direct predictor is missing, then the indirect predictors are relevant. For example, if fever is missing and the patient has not clarified whether they have fever, the machine can ask about it. Even when the patient is reporting that they do not have fever, the LLM can clarify that the information may change:

“You are not reporting fever but we are in early stages of the disease and fever is still probable later, given your current symptoms. In our analysis, we keep in mind that fever may emerge.”

A patient may find it informative that fever is still likely. This may help in his/her management of the disease. In addition, this type of statement clarifies that the model is imputing the value of fever. Transparency in how the machine thinks through the symptoms is important in gaining the trust of the patient. Alternatively, the machine can use its internal inferences to tell the user:

“It has been more than 3 days since onset of your symptoms. You did not mention fever. We have assumed that you do not have fever but if the situation changes, please return as it may change our advice.”

The point is that LLM conversations can reduce missing values and improve accuracy of the model. In fact, the Causal Network provides guidance on where the LLM should look for more clarity. When direct variables are missing, it should verify the presence/absence of indirect variables that allow imputation of the missing value. In multi-turn conversations with the patient, the dialogue manager has to decide when the conversation has been sufficient to make the needed inference. Then, and only then, the intake conversation is concluded. Our data suggests that the conversations should continue until the LLM has verified variables that could change the advice of the system.

This paper has reported on accuracy of using a causal network to guide and train an LLM for engaging patients at home with symptoms of possible COVID-19. The discussion of generalizability beyond COVID-19 is speculative, but an example can help readers understand potential adaptations to other diseases. Consider the outbreak of childhood pneumonia. Wu and colleagues report a Causative Bayesian Network organized from knowledge of experts and validated on pathogens for childhood pneumonia [34]. The model is quite complex, involving various clinical signs, many symptoms, laboratory test results, and epidemiological pathways of the infection. A conversational AI system could help put this model in use. Unfortunately, conversational AI systems rely on the disclosed information, and do not currently probe for unreported variables. Thus, if one of the many laboratory tests (e.g., Xray) has not been ordered or completed, the conversational LLM does not probe further for the predictors of X-ray results. If our study is any indication, such failure to explore will lead to drop in accuracy of the diagnosis of childhood pneumonia. Our study not only warns against failure to explore but also shows exactly what should be explored further: the indirect predictors which have a mediated impact through the missing variable (Xray results) on the outcome (childhood pneumonia). In this manner, Wu and colleagues Causative Bayesian Network becomes a dialogue manager and sets the priority for next topic raised by the LLM.

## Conclusion

A causal network can guide LLMs to explore sufficiency of the disclosed information. When key information is missing, LLMs should probe further for disclosure of missing direct causes. When one or more direct variables cannot be reported, then LLMs should ask for indirect causes that have a mediated impact through the missing information.
